# Versorgungszustand von Patienten vor Zuweisung an ein universitäres Wundzentrum

**DOI:** 10.1007/s00105-021-04759-8

**Published:** 2021-01-28

**Authors:** Cornelia Erfurt-Berge, Melanie Michler, Regina Renner

**Affiliations:** 1 Wundzentrum DDG/ICW, Hautklinik Universitätsklinikum Erlangen, Friedrich-Alexander-Universität Erlangen-Nürnberg (FAU), Ulmenweg 18, 91054 Erlangen, Deutschland; 2 Zentralbereich Medizin: Struktur‑, Prozess- und Qualitätsmanagement, Universitätsklinikum Tübingen, Hoppe-Seyler-Str. 6, 72076 Tübingen, Deutschland

**Keywords:** Versorgungsstruktur, Ulcus cruris, Basisdiagnostik, Primärversorgung, Versorgungsqualität, Care structure, Leg ulcer, Basic diagnostics, Primary care, Quality of care

## Abstract

**Hintergrund und Fragestellung:**

Die Versorgungswege von Patienten mit chronischen Wunden sind häufig sehr langwierig. Dies kann zu einer verminderten Versorgungsqualität und zu einer verspäteten Diagnose der eigentlichen Ursache führen. Gleichzeitig existieren zertifizierte Einrichtungen für diese Patientengruppe. Die vorliegende Arbeit untersucht mögliche Gründe für eine verzögerte Zuweisung an diese Zentren und ob eine spezifische Patientenauswahl an universitäre Zentren gelangt.

**Patienten und Methoden:**

Durch eine retrospektive Auswertung der Patientendatensätze zum Zeitpunkt der Erstvorstellung im zertifizierten Wundzentrum wurden Variablen zum Versorgungszustand vor der universitären Vorstellung analysiert.

**Ergebnisse:**

Es konnten Datensätze von 177 Patienten ausgewertet werden (53 % weiblich, 47 % männlich). Die Altersspanne lag zwischen 27 und 95 Jahren. Die mittlere Bestandsdauer der Wunde betrug 22 Monate. Eine Gefäßdiagnostik war im Vorfeld in 32 % (arterielle Diagnostik) bzw. 36 % (phlebologische Diagnostik) durchgeführt worden. Eine Gewebeprobe war in 9 % der Fälle entnommen worden, v. a. bei Patienten mit > 24 Monaten bestehender Wunde. In nur 45 % der Fälle stimmte die externe Diagnose mit der im Wundzentrum abschließend gestellten Diagnose überein.

**Diskussion:**

Die Versorgungssituation von Patienten mit chronischen Wunden außerhalb spezialisierter Versorgungsstrukturen ist als unzureichend anzusehen. Eine frühzeitige Versorgung nach etablierten Standards in Diagnostik und Therapie sowie zeitnahe Überweisung bei stagnierendem Verlauf an eine Spezialsprechstunde sind anzustreben.

## Abstract (en)

**Background and objectives:**

Health care for patients with chronic wounds is often protracted. This can result in decreased quality of care or delayed diagnosis of the actual cause of disease. Concurrently, there are already certified facilities for these patients. The present work investigates possible reasons for delayed referral and whether a specific selection of patients is sent to these university-based centres.

**Patients and methods:**

A retrospective patient data chart review at the point of first admission to the certified wound centre was performed to identify concerning variables about the standard of care before university presentation.

**Results:**

Records of 177 patients were analysed (53% women, 47% men; patient age range 27–95 years). The mean duration of the wound was 22 months. Vascular diagnostics had already been performed in 32% (arterial diagnostics) and 36% (phlebological diagnostics), respectively. A tissue sample had been analysed in 9% of cases, especially when wound duration exceeded > 24 months. In only 45% of cases was the external diagnosis in accord with the final diagnosis in the wound centre.

**Discussion:**

The health care situation for patients with chronic wounds outside of specialised care structures is insufficient. Early and standardized diagnostics and therapy and a reasonable admission to specialised centres is desired.

In Deutschland leiden etwa 800.000 Menschen an einer chronischen Wunde [1]. Eine Wunde, die nach 8 Wochen nicht abgeheilt ist, gilt als chronisch. Liegt eine chronisch venöse Insuffizienz, eine periphere arterielle Verschlusskrankheit oder ein Diabetes mellitus als Ursache der Ulzeration vor, kann die Wunde von Beginn an als chronisch angesehen werden [2]. Die Versorgung von Patienten mit chronischen Wunden ist kosten-, personal- und zeitintensiv [3]. Dabei ist davon auszugehen, dass frühzeitige Therapie und interprofessionell sowie interdisziplinär strukturierte Behandlungskonzepte zu einer schnelleren Abheilung und damit Kostensenkung führen können [4].

## Hintergrund und Fragestellung

Ziel dieser Arbeit ist es, die präklinische Versorgungssituation von Patienten mit chronischen Wunden zu analysieren und herauszufinden, welches Patientenklientel zur weiteren Behandlung an ein universitäres Wundzentrum überwiesen wird.

## Studiendesign und Untersuchungsmethoden

### Untersuchte Patientengruppe

Es erfolgte eine retrospektive Datenanalyse anhand der medizinischen Dokumentation zum Zeitpunkt der Erstvorstellung eines Patienten im Wundzentrum Dermatologie der Hautklinik des Universitätsklinikums Erlangen. Insgesamt konnten Datensätze von 244 Patienten im Beobachtungszeitraum 08/2015 bis 12/2017 identifiziert werden, die sich erstmals oder nach mehr als 12 Monaten wieder im Wundzentrum Dermatologie vorstellten. Um die verschiedenen Diagnosen einzugrenzen, wurden nur Patienten mit Ulcus cruris unterschiedlicher Genese und neuropathischem Fußulkus für die Analyse herangezogen. Patienten mit Dekubitus, peristomalen Ulzerationen oder ulzerierten Malignomen wurden ausgeschlossen, ebenso Patienten mit Ulzerationen an ungewöhnlichen Lokalisationen (z. B. erosive pustulöse Dermatose der Kopfhaut). Final erfüllten 177 Datensätze diese Auswertungskriterien.

### Erfassung der Variablen

Es wurde anhand der aktuellen Literatur [5] ein Erfassungsbogen mit den verschiedenen Variablen konzipiert. Aufgrund der retrospektiven Datenauswertung und teils fehlender Dokumentation wurde der Erfassungsbogen entsprechend angepasst. Gesammelt wurden hierbei Variablen zu demografischen Angaben, Diagnose, Zuweisungsgrund, Wundsituation, bisheriger ärztlicher Betreuung, Versorgungssituation im häuslichen Umfeld sowie Diagnostik und Therapie im Vorfeld der universitären Vorstellung (Tab. 1). Als Bewertungskriterien für eine ausreichende präklinische Diagnostik und Therapie wurden anhand bekannter Zielkriterien aus der Literatur [5] für die geplante Analyse folgende Kriterien definiert:

**Table 1:** 

Epidemiologie	Geschlecht
	Geburtsdatum und Alter am Tag der Erstvorstellung
	Wohnort, Stadttyp und Distanz zum Wundzentrum (in km)
	Wohnsituation (zu Hause oder im Pflegeheim)
	Datum der Erstvorstellung
Diagnose	Überweisungsdiagnose
	Diagnose und ICD-Code im Wundzentrum
	Übereinstimmung der Diagnosen
Zuweisungsgrund	Anlass: Patient kommt aus eigener Motivation, auf Anraten des Pflegediensts, Konsil, Über- oder Einweisung
	Wiederkehrer nach > 12 Monate zurückliegender Erkrankung
Wundsituation	Bestandsdauer der Wunde (in Monaten) und Rezidiv
	Wundfläche (summiert, in cm^2^)
	Verlauf
	Weitere Therapieempfehlung (ambulant, stationär, teilstationär) nach Erstvorstellung
Bisherige ärztliche Betreuung	Hausarzt oder Internist
	Hautarzt
	Anderer „Wundfacharzt“ (Chirurg, Gefäßchirurg, Angiologe)
	Zuletzt stationäre Behandlung (<12 Monate)
	Behandlung in einem anderen Wundzentrum
Versorgungssituation im häuslichen Umfeld	Ambulanter Pflegedienst
	Wundmanager oder Homecarer
	Verbandswechselnde Person
	Verbandswechsel Häufigkeit
	Klinikinterner Sozialdienst – Konsil beauftragt
Bisherige Diagnostik	Untersuchung der Venen
	Untersuchung der Arterien
	Histologische Untersuchung
	Abstrich
Bisherige Therapie	Moderne Wundauflage
	Hauttransplantation oder Débridement
	NPWT
	Kompressionstherapie
	Orthopädischer Schuh
	Operation Arterien (beispielsweise PTA, Bypass, Angioplastie)
	Operation Venen (beispielsweise Venenstripping, Fasziotomie, Sklerosierung)

*NPWT* Unterdruckwundtherapie

Eine ausreichende präklinische Diagnostik bzw. Therapie wird als erfolgt gewertet, wenn vor der Erstvorstellung im klinischen Wundzentrum

- die arterielle und phlebologische Basisdiagnostik (Tasten der Fußpulse, Doppler-Untersuchung, Bestimmung des Knöchel-Arm-Druck-Index [KADI], farbkodierte Duplexsonographie) bei Neuauftreten einer Ulzeration durchgeführt bzw. bei einer Ulzeration > 12 Monate Bestandsdauer aktualisiert wurde,
- eine histologische Gewebeuntersuchung innerhalb von 6 Monaten Bestandsdauer der Ulzeration, insbesondere bei unklarer Pathogenese, erfolgt ist,
- Patienten mit der Diagnose Ulcus cruris venosum als kausaltherapeutischen Behandlungsansatz eine Kompressionstherapie erhalten haben,
- Patienten mit neuropathischem Fußulkus als kausaltherapeutischen Behandlungsansatz eine Entlastungstherapie z. B. durch angepasstes Schuhwerk erhalten haben.

### Statistik

Es erfolgte eine Datenauswertung mit der Statistiksoftware IBM SPSS Statistics 2‑0. Die Patienten wurden nach verschiedenen Kriterien gruppiert, um Rückschlüsse auf die Versorgungsqualität der einzelnen Subgruppen zu ziehen. Die Mittelwerte der Gruppen wurden mit statistischen Tests (Chi-Quadrat-Test, U‑Test nach Mann und Whitney, H‑Test nach Kruskal und Wallis Phi) auf einem Signifikanzniveau von *p* < 0,05 verglichen.

## Ergebnisse

### Epidemiologie

Es konnten insgesamt 177 Patientendatensätze ausgewertet werden. Die Altersspanne lag dabei zwischen 27 und 95 Jahren (Median 73 Jahre). Der Anteil an weiblichen Patienten war mit 53 % leicht erhöht gegenüber den Männern. Nur ein geringer Anteil (<5 %) der Patienten war in einer stationären Betreuungseinrichtung untergebracht. Im Durchschnitt wohnten die Patienten 43 km vom universitären Wundzentrum entfernt.

### Wundparameter

Die mittlere Wundfläche betrug 43,4 cm^2^ (Median 14,5 cm^2^), wobei eine sehr große Schwankungsbreite (0,25–600 cm^2^) beobachtet wurde und auch Patienten mit Gamaschenulzera vorstellig waren. Die mittlere Bestehensdauer der chronischen Wunde betrug 22 Monate (Median 8,5 Monate) und reichte von weniger als 4 Wochen bis mehrere Jahre zum Zeitpunkt der Erstvorstellung. Die längste durchgängige Bestehensdauer wurde von einem Patienten anamnestisch mit 20 Jahren angegeben. In 41 % der Fälle handelte es sich um eine Rezidiverkrankung, wobei diese Information nur eingeschränkt aus der retrospektiven Datenanalyse verifiziert werden konnte.

### Zuweisungswege und Weiterbehandlung

Insgesamt 62 % der Patienten stellten sich mit Über- oder Einweisung durch den behandelnden Arzt vor. Dabei erfolgte diese größtenteils durch den Hausarzt (87 % der Ein- bzw. 71 % der Überweisungen). Weitere 23 % suchten das Wundzentrum aus eigener Motivation auf. Die weiteren Zuweisungen erfolgten als Konsile aus externen Kliniken oder auf Anraten eines Pflegedienstes; 82 % der Patienten gaben an, zur Wundbehandlung in regelmäßiger Betreuung beim Hausarzt zu sein, 35 % suchten deshalb zusätzlich oder ausschließlich einen Hautarzt und 50 % einen anderen Facharzt mit Wundschwerpunkt (Gefäßchirurg, Angiologe) auf. Im Schnitt wurden die Patienten bei 2 Fachdisziplinen vorstellig, ehe sie das dermatologische Wundzentrum aufsuchten.

In 50 % der Erstvorstellungen erfolgte im Anschluss die stationäre Aufnahme des Patienten in die Hautklinik. Etwa 33 % der Patienten wurden nach Erstvorstellung zunächst ambulant oder teilstationär an die Klinik angebunden. In 17 % erfolgte eine Therapieempfehlung an den niedergelassenen Kollegen zur Weiterbehandlung; 25 % aller Patienten wurde zusätzlich die Anbindung an einen Pflegedienst oder Homecare-Dienst angeraten und in 18 % aller Fälle ein Kontakt zum klinikinternen Sozialdienst hergestellt.

### Diagnostik und therapeutische Maßnahmen vor Vorstellung im Wundzentrum

**Figure 1:**
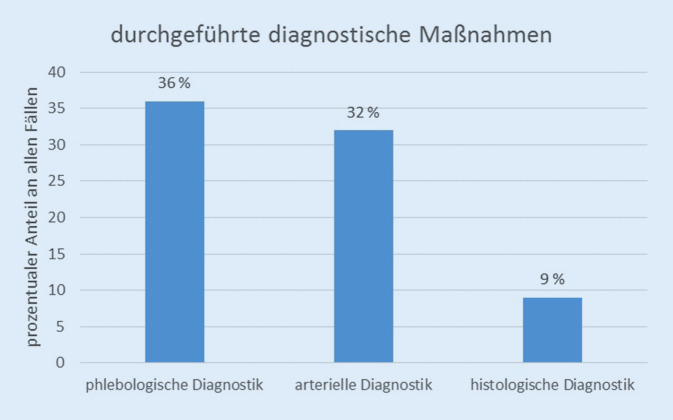

**Figure 2:**
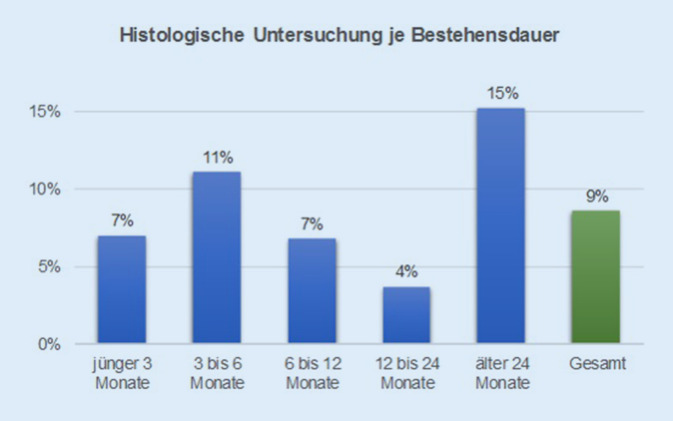

Die Abb. 1 zeigt die Häufigkeit einer im Vorfeld der universitären Vorstellung durchgeführten Basisdiagnostik der arteriellen Versorgungssituation der Unterschenkel (mindestens KADI-Messung, ggf. weiterführende Maßnahmen) oder des phlebologischen Status (Duplexsonographie). Eine Probebiopsie war vorab bei insgesamt 9 % der Patienten entnommen worden. Gehäuft erfolgte dies in der Gruppe der seltenen Diagnosen (31 % der Patienten in dieser Gruppe). Die Abb. 2 zeigt, dass die Gewebeentnahme bevorzugt bei Wundheilungsstörungen > 24 Monaten erfolgte.

**Figure 3:**
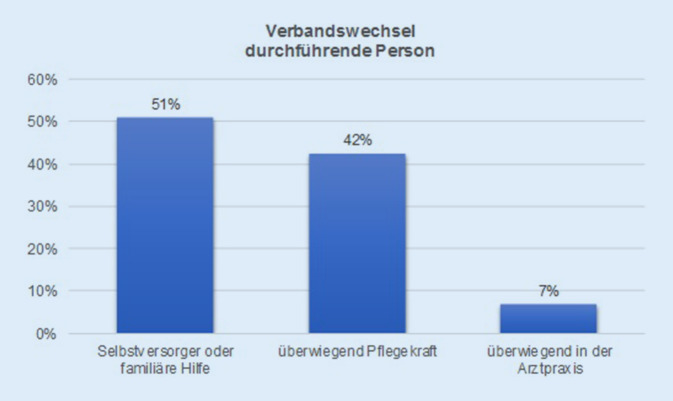

Es wurden 77 % der Patienten im Sinne einer hydroaktiven Wundtherapie mit sog. modernen Wundauflagen behandelt. In 31 % der Fälle war bereits ein ambulanter Pflegedienst an der Wundversorgung beteiligt; 27 % der Patienten waren allein oder zusätzlich (12 %) durch einen Wundexperten eines Homecare-Unternehmens mit betreut; 51 % der Patienten versorgten sich größtenteils selbst. Die Abb. 3 zeigt, dass der Verbandswechsel überwiegend durch den Patienten selbst oder einen Angehörigen übernommen wurde. In 49 % der Fälle erfolgt die Wundbetreuung durch eine medizinisch geschulte Person. Aus der Gruppe der Patienten mit Ulcus cruris venosum (UCV) war bei insgesamt 77 % eine Kompressionstherapie vorab verordnet gewesen. Bei Erstvorstellung im Wundzentrum zeigte sich in 40 % dieser Fälle, dass die verordnete Kompressionstherapie unzureichend angewendet bzw. angelegt worden war. Eine Druckentlastung bei neuropathischem Fußulkus war in 54 % (*n* = 7) dieser Patienten verordnet worden. Davon waren 43 % (*n* = 3) nicht korrekt angepasst. Von den Patienten mit UCV hatten bereits 32 % im Vorfeld phlebochirurgische Eingriffe wie Sklerosierungen oder Venenstrippings sowie von den Patienten mit arteriell bedingtem Ulcus cruris 57 % gefäßchirurgische Eingriffe wie Ballondilatationen oder Bypassoperationen erhalten; 23 % aller Patienten hatten bereits in der Vorgeschichte ein chirurgisches Débridement oder eine Hauttransplantation erhalten, bei 10 % der Patienten war bereits 1‑mal eine Unterdruckwundtherapie (NPWT) durchgeführt worden.

### Diagnosen

**Figure 4:**
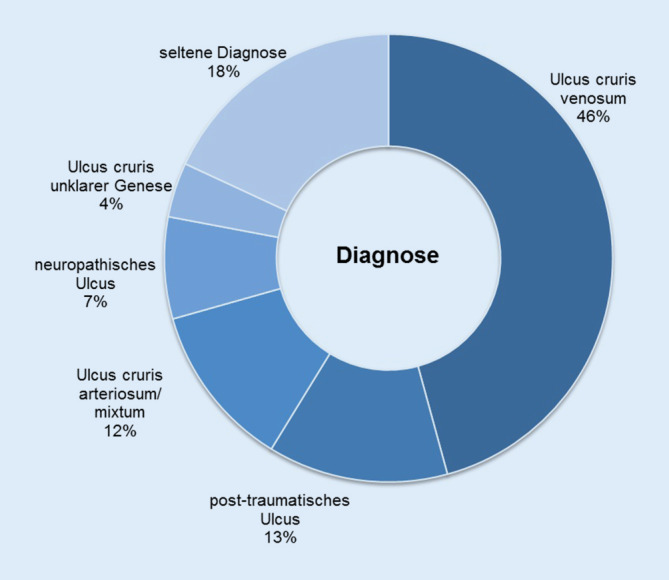

Die Abb. 4 zeigt die unterschiedlichen Abschlussdiagnosen der Patienten, gestellt durch die Behandler im universitären Wundzentrum. Von 144 Patienten konnte anhand von Arztbriefen oder Überweisungsscheinen eine externe Diagnosebezeichnung ermittelt werden. In 45 % dieser Fälle stimmte die externe Diagnose mit der durch die Diagnostik im Wundzentrum abschließend gestellten Diagnose überein. Von den 144 ermittelbaren externen Diagnoseangaben waren 34,0 % als ungenau einzustufen wie beispielsweise die Angabe „Ulcus cruris“ oder „Wundheilungsstörung“. Im Falle einer abweichenden Diagnose wurde in 31,3 % der Fälle eine seltene Ursache der Ulzeration gefunden. Zu den mit einem Gesamtanteil von 18 % aller Fälle relativ häufig vertretenen seltenen Diagnosen zählten dermatospezifische Wundursachen wie Pyoderma gangraenosum, ulzerierte Necrobiosis lipoidica oder Kalziphylaxie. Von den Patienten mit Diagnoseänderung im Laufe der Behandlung im Wundzentrum hatten 27,8 % jeweils nur eine vaskuläre Diagnostik (KADI-Messung oder Duplexsonographie) erhalten, 16,6 % hatten beide Untersuchungen, 55,6 % keine vaskuläre Diagnostik in den letzten 12 Monaten vor Erstvorstellung erhalten. Des Weiteren hatten 91,7 % aus dieser Gruppe bislang noch keine histologische Untersuchung erhalten.

## Diskussion

In Deutschland existieren bereits zahlreiche zertifizierte ambulante und stationäre Wundzentren. Dennoch stellen sich Patienten dort oft erst nach längerem Krankheitsverlauf vor. Stoffels-Weindorf et al. [6] konnten in einer monozentrischen Untersuchung zeigen, dass ungewöhnliche Ulkusursachen, wie z. B. eine Vaskulitis, Rezidiverkrankung oder starke Schmerzen, einen Triggerfaktor für eine frühzeitige Vorstellung in einem Wundzentrum darstellen. Der Anteil an Patienten mit seltener Ursache der chronischen Wunde lag in dieser Untersuchung aus einem dermatologischen Zentrum bei 20 %. Es ist zu diskutieren, wie die Angebote zertifizierter Zentren an die Erstversorger, Hausarztpraxen oder ambulante Pflegedienste herangetragen und für die Patienten eine schnellere Zuweisung realisiert werden kann. Hierzu ist es erforderlich, Zuweisungswege und -gründe besser zu analysieren. Auch in unserer Auswertung zeigte sich eine mittlere Bestandsdauer von fast 2 Jahren vor Erstzuweisung. Die Gründe hierfür sind vielfältig. Eine mögliche Erklärung sind fehlende Mobilität und Möglichkeit des Patienten selbst, in ein empfohlenes Zentrum zu gelangen. Aus eigener Erfahrung scheuen viele Patienten auch den Wechsel und progressive Maßnahmen. Für die Autoren überraschend war die Tatsache, dass der Großteil der behandelten Patienten im Schnitt eher kleine bis mittelgroße Wundflächen aufwies. Durchaus auffällig ist der mit über 40 % erhöhte Anteil an Rezidiverkrankungen unter den Patienten zum Zeitpunkt der Erstvorstellung. Es lässt sich vermuten, dass hier der Vorbehandler aufgrund der Erfahrung bei primärer Erkrankung vermutlich schneller zu einer Zuweisung im Falle eines Rezidivs riet. Nicht detailliert analysieren lässt sich anhand der retrospektiv erhobenen Daten, ob die Überweisung ins Wundzentrum immer auf Betreiben des ärztlichen Behandlers oder des Pflegeteams erfolgte. Mit 23 % gab ein relativ großer Anteil der Patienten an, aus eigener Motivation vorstellig zu werden und eine Zweitmeinung zu wünschen.

In dieser Untersuchung lag die mittlere Entfernung vom Wohnort zum Wundzentrum bei 43 km. Große Wundflächen und Bestehensdauer der Ulzeration seit > 6 Monaten konnten als mögliche Motivation für eine längere Anreise zum Wundzentrum identifiziert werden. Auswertungen aus vergleichbaren Zentren in Deutschland zeigen ähnliche Ergebnisse [7]. Es kann vermutet werden, dass die Versorgung in einer infrastrukturell optimalen Metropolregion wie Erlangen-Fürth-Nürnberg besser als in ländlichen Regionen ist. Zudem stehen den Patienten aus den umgebenden ländlichen Regionen mehrere Wundspezialisten als Ansprechpartner zur Verfügung. Dennoch zeigt die auffällig lange Wunddauer bis zur Erstvorstellung unserer Klientel die Notwendigkeit einer frühzeitigeren Zuweisung auf, um zielführend und kostensenkend zu arbeiten.

Die bei Zuweisung an das Wundzentrum vorliegende Diagnose musste in über der Hälfte der Fälle revidiert werden. In 5 % aller Fälle war keine Überweisungsdiagnose eruierbar. Einschränkend muss diskutiert werden, dass im Einzelfall ggf. aufgrund von Kommunikationsfehlern (z. B. fehlende Angabe auf der Überweisung) nähere Informationen zur Arbeitsdiagnose des Vorbehandlers fehlten und somit nicht in die Auswertung einfließen konnten. Aufgrund der Beobachtung, dass eine vaskuläre Basisdiagnostik bei der untersuchten Kohorte nicht standardmäßig und regelhaft erfolgte, muss diskutiert werden, ob und wenn ja aus welchen Gründen keine Arbeitsdiagnose im Zeitraum der Vorbehandlung erstellt wurde. Denkbar wäre hier beispielsweise eine lange Wartezeit auf fachärztliche Diagnostikuntersuchungen. Kürzlich veröffentlichte Empfehlungen [6] zur Verbesserung der Versorgungsstruktur für Menschen mit chronischen Wunden in Deutschland zeigen genau diese Defizite auf und empfehlen den Aufbau eines Versorgungsnetzwerkes für die Behandlung dieser Patienten. Sollte ein behandelnder Arzt nicht über bestimmte Diagnostikmöglichkeiten verfügen, so ist anzustreben, den Diagnoseschritt durch entsprechende Netzwerkpartner zu erreichen. Die Notwendigkeit einer frühzeitigen adäquaten Diagnose zur Erstellung eines Therapieplanes wird vom Expertenrat Strukturentwicklung Wundmanagement betont [6]. Insbesondere trifft dies zu, wenn wie im Falle unseres untersuchten Wundzentrums auch eine Vielzahl an Patienten mit selteneren Ursachen chronischer Wunden vorstellig wird. Hier kann die notwendige Expertise nur an ausgewählten Zentren eingeholt werden. Darüber hinaus müssen erkrankungsspezifische Therapiemaßnahmen eingeleitet werden [8]. Aufgrund der limitierten Datenmenge zeigte sich in unserer Auswertung kein Zusammenhang zwischen seltener Ursache der Wunde und dadurch bedingter frühzeitiger Vorstellung im Zentrum.

Der Anteil der Patienten, die den Verbandswechsel selbstständig oder durch Angehörige durchführten, war mit 51 % auffallend hoch. Allein 31 % der Patienten wurden durch einen ambulanten Pflegedienst betreut. Dies ist insofern relevant, da in anderen Studien gezeigt werden konnte, dass für die korrekte Anlage eines Kompressionsverbandes eine besondere Schulung notwendig ist und dies häufig nicht vom Patienten selbst durchgeführt werden kann [9, 10]. Insbesondere patienten- und anwenderfreundliche Mehrkomponentensysteme sind noch unzureichend verbreitet, auch wenn sie beispielsweise die Behandlung eines Selbstversorgers deutlich optimieren könnten [11]. Für alle Systeme gleichermaßen notwendig ist aber eine adäquate Patientenedukation und Anleitung, insbesondere wenn der Patient darauf besteht, Verbandswechsel eigenständig durchzuführen. Eine monatelange inadäquate Behandlung mit unzureichend angelegten Kurzzugbinden sollte unbedingt vermieden werden [12].

Die in unserer Analyse erhobenen Daten von etwa 75 % verordneter Kompressionstherapie bei Diagnose eines Ulcus cruris venosum deckt sich mit Ergebnissen früherer Untersuchungen ebenso wie die Tatsache, dass trotz Verordnung die Kompressionstherapie häufig nicht adäquat angewendet wird [9]. Untersuchungen an Krankenkassendaten zeigen sogar noch deutlich niedrigere Raten von knapp 40 % hinsichtlich Verordnung kausaltherapeutischer Kompression bei UCV [13]. Auch Untersuchungen in anderen Gesundheitssystemen wie beispielsweise in Großbritannien zeigten ein heterogenes Vorgehen statt strukturierter Versorgung nach Standards [14]. Große Wundfläche und höheres Alter wurden zusätzlich als Risikofaktoren für eine verlängerte Wundheilung erkannt [15]. Durch frühzeitige Optimierung von Diagnostik und Therapie können dem Patienten nicht nur lange Krankheitsdauer, Schmerzen und Komplikationen erspart werden [16]. Durch die Vermeidung langer Behandlungszeiten und stationärer Aufnahmen werden zudem auch Kosten gespart [17]. Auch wenn nationale und internationale Fachgesellschaften bereits mehrfach Standards und Empfehlungen zur Behandlung von Patienten mit chronischen Wunden erarbeitet haben [6, 18], so fehlt weiterhin die Implementierung v. a. im präklinischen Bereich. Die Versorgungssituation von Patienten mit chronischen Wunden außerhalb spezialisierter Versorgungsstrukturen ist daher weiterhin als unzureichend anzusehen.

## Fazit für die Praxis

- Frühzeitige zielgerichtete Diagnostik verkürzt die Behandlungsdauer und verbessert die Lebensqualität der Patienten mit chronischen Wunden. So können rechtzeitig kausaltherapeutische Maßnahmen eingeleitet werden.
- Die Behandlung von Patienten mit chronischen Wunden sollte dabei möglichst anhand von Standards und Empfehlungen der Fachgesellschaften erfolgen.
